# Data on the phosphorylation of p38MAPK and JNK induced by chlorpyrifos in *Drosophila melanogaster*

**DOI:** 10.1016/j.dib.2016.08.033

**Published:** 2016-08-24

**Authors:** J.E.S. Batista, L.R. Sousa, I.K. Martins, N.R. Rodrigues, T. Posser, J.L. Franco

**Affiliations:** Oxidative Stress and Cell Signaling Research Group. Universidade Federal do Pampa, Campus São Gabriel, São Gabriel, RS, Brazil

**Keywords:** Organophosphate compounds, Cell signaling, Stress response, Biomarkers, Mechanisms of toxicity

## Abstract

Exposure to organophosphate compounds, such as chlorpyrifos, has been linked to disturbances on cell signaling pathways. Mitogen activated protein kinases (MAPK) are a family of protein kinases involved in a range of cellular processes, including stress response, apoptosis and survival. Therefore, changes in the activation state of these kinases may characterize key mechanisms of toxicity elicited by xenobiotics. Here we report data on the phosphorylation of p38MAPK and JNK, members of the MAPK family, in *Drosophila melanogaster* exposed to chlorpyrifos, as characterized by western blotting assays.

**Specifications Table**

**Table:** 

Subject area	*Biology*
More specific subject area	*Biochemistry/Toxicology*
Type of data	*Figure*
How data was acquired	*Western blots, chemiluminescence*
Data format	*Raw, analyzed*
Experimental factors	*Flies were exposed during 24* *h with chlorpyrifos diluted in 1% sucrose*
Experimental features	*Changes in MAPK phosphorylation were analyzed in flies exposed or not to chlorpyrifos*
Data source location	*Universidade Federal do Pampa, São Gabriel, RS, Brazil*
Data accessibility	*Data provided in article.*

**Value of the data**

• The data describe changes in the phosphorylation of p38MAPK and JNK after exposure of Drosophila to organophosphate compound, chlorpyrifos, by means of western blotting techniques.
• The data can be used as a reference regarding the phosphorylation state of these protein kinases as a response to organophosphate compound poisoning in a complex biological system *in vivo*.
• Taking into consideration the involvement of mitogen activated protein kinases (MAPK) in a range of cellular processes, including stress response, apoptosis and survival [1], following changes in the phosphorylation state of these proteins may be of wide interest to researchers investigating biomarkers and mechanisms of toxicity elicited by environmental toxicants.

## 1 Data

**Fig. 1:**
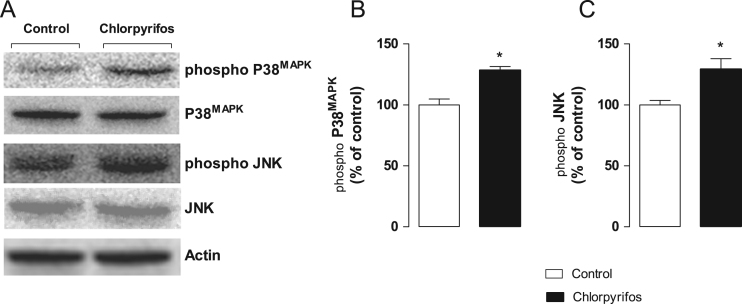
Representative immunoblots of total and phosphorylated forms of p38MAPK and JNK as well as β-actin are depicted in Fig. 1A. The densitometric analysis of immunoreactive bands revealed that flies exposed to CP under our experimental conditions presented a significant increase (*p*<0.05) in the expression of phosphorylated forms of p38MAPK and JNK (Fig. 1B and C, respectively). The protein levels of β-actin as well as total forms of MAPK were unchanged (Fig. 1A).

We evaluated the expression of total and phosphorylated forms of two major protein kinases belonging to the MAPK family: p38MAPK and JNK, in flies exposed during 24 h to chlorpyrifos (0.75 µg/ml in 1% sucrose). Control flies received 1% sucrose only. It was observed a significant increase (*p*<0.05) in the phosphorylation of p38MAPK (Fig. 1B) and JNK (Fig. 1C) in flies exposed to chlorpyrifos when compared to control. No changes in the content of total forms of p38MAPK and JNK, as well as β-actin (loading control) were observed in both treated and control flies (Fig. 1A).

## 2 Experimental design, materials and methods

All experimental procedures, including Drosophila stock and culture, sample preparation, western blotting and statistical analysis were performed according to previously published methods [2], [3], [4].

### 2.1 *Drosophila melanogaster* treatment

Adult female flies (1–4 days) were left, overnight, in glass tubes containing filter paper soaked in 1% sucrose for acclimation. Subsequently, 30 flies per group were kept in glass tubes containing filter paper soaked with 250 µL of each treatment solution. The experimental groups were: Control (received 1% sucrose solution only) and Chlorpyrifos (CP) 0.75 ppm (diluted in 1% sucrose solution). The CP concentration used here was based on the LC_50_ (24 h) in adult female flies (1.182 ppm), which was previously determined by our group [4]. Each experiment was repeated at least three times (n=3). After treatments were finished, samples were prepared for analysis of MAPK phosphorylation as described previously [3], [4].
