# Supplementary material for: Data on the phosphorylation of p38MAPK and JNK induced by chlorpyrifos in Drosophila melanogaster
Source: Data Brief. 2016 Aug 24;9:32–4. doi: 10.1016/j.dib.2016.08.033 (PMC5011164; doi:10.1016/j.dib.2016.08.033)
Supplement: Supplementary file 1 — Supplementary material [file mmc1.docx]

**UNIVERSIDADE FEDERAL DO PAMPA**

**CAMPUS SÃO GABRIEL**

## Av. Antonio Trilha, 1847, Centro. São Gabriel, RS, Brazil 97300-000

25^th^ July 2016

**Conflict of interest declaration**

Dear Editor,

All authors of the submitted manuscript entitled *"* **Data on the phosphorylation of p38MAPK and JNK induced by chlorpyrifos in *Drosophila melanogaster*”** from authorship of Batista et al., are in agreement with submission.

All authors declare no conflict of interest. A statement on this regard was included in the main text of the manuscript.

Here we report data on the phosphorylation of p38MAPK and JNK, members of the MAPK family, in *Drosophila melanogaster* exposed to chlorpyrifos, an organophosphate compound widely used in agriculture and as a home pesticide.

Sincerely,

Jeferson Luis Franco, Ph.D

Universidade Federal do Pampa

Campus São Gabriel

São Gabriel – RS (97.300-000)

[jefersonfranco@unipampa.edu.br](mailto:jefersonfranco@unipampa.edu.br)
